# Matrix Metalloproteinase 9 Exerts Antiviral Activity against Respiratory Syncytial Virus

**DOI:** 10.1371/journal.pone.0135970

**Published:** 2015-08-18

**Authors:** Abdoulaye J. Dabo, Neville Cummins, Edward Eden, Patrick Geraghty

**Affiliations:** Mount Sinai St. Luke’s Medical Center, Mount Sinai Health System, Division of Pulmonary, Critical Care and Sleep Medicine, New York, NY, United States of America; Imperial College London, UNITED KINGDOM

## Abstract

Increased lung levels of matrix metalloproteinase 9 (MMP9) are frequently observed during respiratory syncytial virus (RSV) infection and elevated MMP9 concentrations are associated with severe disease. However little is known of the functional role of MMP9 during lung infection with RSV. To determine whether MMP9 exerted direct antiviral potential, active MMP9 was incubated with RSV, which showed that MMP9 directly prevented RSV infectivity to airway epithelial cells. Using knockout mice the effect of the loss of *Mmp9* expression was examined during RSV infection to demonstrate MMP9’s role in viral clearance and disease progression. Seven days following RSV infection, *Mmp9* ^-/-^ mice displayed substantial weight loss, increased RSV-induced airway hyperresponsiveness (AHR) and reduced clearance of RSV from the lungs compared to wild type mice. Although total bronchoalveolar lavage fluid (BALF) cell counts were similar in both groups, neutrophil recruitment to the lungs during RSV infection was significantly reduced in *Mmp9* ^-/-^ mice. Reduced neutrophil recruitment coincided with diminished RANTES, IL-1β, SCF, G-CSF expression and p38 phosphorylation. Induction of p38 signaling was required for RANTES and G-CSF expression during RSV infection in airway epithelial cells. Therefore, MMP9 in RSV lung infection significantly enhances neutrophil recruitment, cytokine production and viral clearance while reducing AHR.

## Introduction

Matrix metalloproteinase 9 (MMP9) is a zinc-binding endopeptidase that degrades multiple extracellular matrix molecules [1]. Persistent MMP9 signaling is associated with tissue remodeling [2] and the progression of many lung diseases including chronic obstructive pulmonary disease (COPD) [2], asthma [3], airway infections [4, 5] and idiopathic pulmonary fibrosis [6]. MMP9 is produced by a range of cells in the respiratory tract and also plays several roles in modulating immune responses [7]. MMP9 can activate [8, 9] but equally inactivate several cytokines and chemokines [1]. MMP9 expression is required for neutrophil migration in an influenza infection mouse model and also regulated viral replication [10]. Increased MMP9 levels are detected at the time of respiratory syncytial virus (RSV) infection, a virus frequently reported in infants, the elderly, immunocompromised patients as well as healthy adults [11]. This increase in lung MMP9 concentration correlates with disease severity [12]. Recently, several investigators have demonstrated that MMPs play a key role during pulmonary viral infection [10, 13–15]. However the functional role of MMP9 during an RSV infection is unknown.

It is estimated that almost everyone has experienced at least one RSV infection as an infant [16] but immunity against RSV infections is uncommon. Therefore, identifying the functional role of host-induced proteases during RSV infection may be beneficial in determining new therapeutic approaches to counter this virus. We recently demonstrated that RSV induces a network of host proteases in mouse airways [13]. Here we choose to investigate MMP9 as it is linked to several RSV-induced disease manifestations, including airway hyperresponsiveness (AHR) and MMP9 is present at high concentrations in neutrophils, which are the major immune cell present in human BALF from RSV patients [17]. Inhibition of MMP9 activity in bronchial epithelial cells was previously reported to prevent syncytia formation and block RSV multiplication [18] but *in vivo* studies are required to demonstrate causality of MMP9’s role during RSV infection.

In this study, *in vivo* profiling of MMP9 gene and protein expression demonstrated that lung MMP9 levels coincides with neutrophil numbers. Interestingly, MMP9 expression was required for neutrophil migration into the lung in response to RSV infection. *In vitro* and *in vivo* assays also determined that MMP9 significantly reduces RSV replication, host cell viability, airway hyperresponsiveness (AHR), body weight loss and enhances RANTES, IL-1β, SCF and G-CSF production during RSV infection. These findings establish some of the diverse roles of MMP9 in the lungs during RSV infection, such as anti-RSV properties and augmenting neutrophil recruitment to the lungs.

## Methods

### Ethics statement

This study was performed in strict accordance with the recommendations in the Guide for the Care and Use of Laboratory Animals of the National Institutes of Health and Institutional Animal Care and Use Committee (IACUC) guidelines. Mount Sinai St. Luke’s Medical Center’s Institutional Animal Care and Use Committee approved the protocol. Animals were monitored several times daily, and any mice exhibiting signs of distress were euthanized. Distress was defined as animals showing one or several of the following characteristics; ruffled fur, arched back, respiratory distress (e.g. gasping), weight loss greater than 25% and any notable unusual behavior. Investigators consulted with veterinarians if animals appeared distressed. All animals were anesthetized by intraperitoneal (IP) injection of a mixture of ketamine and xylazine and euthanized by asphyxiation with carbon dioxide.

### RSV culture

Human RSV strain A2 (ATCC, Manassas, VA; #VR-1540) was cultured and virus titers quantified by performing plaque assays, as previously described [19]. Non-infected cell cultures were processed in the same manner as RSV infected cells and the resulting sample collection was used as a mock control.

### Animal models

*Mmp9* knockout (FVB.Cg-Mmp9^tm1Tvu^/J) mice and their wild type (WT) littermates were purchased from the Jackson Laboratory (Bar Harbor, ME) on a FVB/NJ background. Mice were maintained in a specific pathogen-free facility at Mount Sinai St. Luke’s Medical Center. 8-week-old mice were used at the initiation point for all experiments and each experimental parameter had 10 animals per group. Mice were anesthetized by intraperitoneal injection of a mixture of ketamine and xylazine. Animals were intranasally administered 1x10^6^ plaque forming units (pfu) of RSV or mock. Animals were weighed daily. Bronchoalveolar lavage fluid (BALF) and lung tissue were collected. The lungs underwent pressure-fixation as previously described [19] and in accordance with the American Thoracic Society/European Respiratory Society issued statement on quantitative assessment of lung structure [20]. Immune cell counts and cell types were determined by flow cytometry. Raw data from one representative mouse with gating conditions for neutrophil characterization is shown in S1 Fig. BALF cells were also collected by cytospins and stained with Diff-Quik to visually identify cell populations. Concentration of RSV N (pg) was determined by qPCR in lung homogenates, as previously described [19]. Results are represented as natural log pico grams (pg). Viral titers were also quantified by performing plaque assays on lung homogenates, as previously described [19].

Bone marrow derived macrophages (BMDM) were obtained from mice (WT and *Mmp9* ^-/-^) by flushing mouse tibiae and femurs with ice-cold PBS through a 70 μm-wide cell strainer and seeding cells at 37°C for 2 hours in a humidified atmosphere with 5% CO_2_ and then removing non-adherent cells. Cells were incubated in growth medium (MEM, 10% FBS, 2 mM glutamine, 100 IU/ml of penicillin, 100 μg/ml of streptomycin and 20 ng/ml M-CSF) for 7 days prior to RSV infection.

### Airway responses to methacholine challenge

Airway responses to methacholine (Sigma Chemical, St. Louis, MO) were assessed with the Scireq Flexivent system (Scireq, Montreal, QC, Canada) at 7 days post infection (dpi). Animals were anesthetized with ketamine/xylazine (10 mg/kg) and paralysis was induced with 1 mg/kg pancuronium bromide IP (Sigma). The linear single-compartment model was used to assess total respiratory system resistance (Rn). Methacholine dose responses were determined as previously described [13, 21].

### BALF analysis

MMP9 (EMD MILLIPLEX MAP MMP Magnetic Bead Panels, EMD Millipore, Billerica, MA) and cytokines (IL-13, RANTES, CXCL2, TNF-α, MCP-1, IL-1β, IL-17, IL-10, CXCL1, G-CSF, IL-6 and human IL-8; Bio-Rad Cytokines Magnetic Bead Panels) were measured in BALF and cell culture supernatants using a bead assay (MILLIPLEX MAP MMP Magnetic Bead Panels, EMD Millipore, Billerica, MA) with the Bio-Rad Bio-Plex 200 system (Bio-Rad, Hercules, CA) or with commercially available ELISAs (CXCL5 and SCF; R&D Systems). BALF gelatinase activity was determined by gelatin zymography [13]. Densitometry was performed on MMP9 positive bands and represented densitometry units (DU) measured by pixel intensity of bands, using Bio-Rad Laboratories Image Lab software (version 4.0, build 16).

### Apoptosis measurements

BALF cells were analyzed for apoptotic cells utilizing the LIVE/DEAD cell viability assay from Invitrogen on the Guava easyCyte flow cytometer from Millipore. The apoptotic cells were expressed as a percentage of total BALF cells.

### Cell culture

Monolayers of human small airway epithelial (SAE) cells from healthy subjects directly obtained from Lonza (Walkersville, MD, catalogue # CC-2547) were submerged cultured as previously described [22, 23]. All cells are assayed and test negative for HIV-1, mycoplasma, Hepatitis-B, Hepatitis-C, bacteria, yeast and fungi upon isolation by Lonza. Cells were only used for experiments at passages 3–6 and at a confluency of approximately 70%. SAE cells were transfected by administering silencing RNA (siRNA) specific for p38, MMP9 or negative control scrambled (Scr.) (Qiagen, Gaithersburg, MD). Cells were infected with RSV at a MOI of 0.3 or treated with mock or human MMP9 protein for 24 hours. Human MMP9 (Abcam; ab168863) was activated by incubating with 2mM 4- aminophenylmercuric acetate (APMA; Sigma) at a 9:1 (MMP9:APMA) ratio for 90 minutes at 37°C and inactivated with 2mM EDTA. Active and inactive MMP9 were dialyzed twice in TBS to remove APMA or EDTA prior to treating cells or RSV. RSV was treated with various concentrations of active and inactive MMP9 prior to infecting SAE cells. TCID_50_ assays were performed to determine the quantity of virus following MMP9 treatment. TCID_50_ data was calculated using the method of Reed and Munch [24]. SAE cells were also treated with active and inactive MMP9 alone and TCID_50_ assays were performed.

### Intracellular signaling

Lung tissue protein from mice was homogenized in radio-immunoprecipitation assay (RIPA) buffer, centrifuged at 13,000 x g for 10 minutes and supernatants collected. Lung protein extracts were assayed for myeloperoxidase (MPO) activity using a kit from Cayman Chemical Company (Ann Arbor, MI) as recommended by the manufactures. p-p38 and p38 levels in mouse lung lysates and BMDM were examined using a beads assay with the Bio-Plex 200 system (Bio-Rad). Activity was represented as fluorescent intensity of the phosphorylated proteins over total proteins. Immunoblots were also conducted to determine levels of p-p38 (Thr180/Tyr182) (Catalogue #4511; monoclonal antibody produced from rabbits immunized with synthetic phosphopeptide corresponding to residues surrounding Thr180/Tyr182 of human p38 MAPK; final concentration 1/1000 dilution), p38 MAPK (Catalogue #9212; polyclonal antibody produced from rabbits immunized with synthetic peptide corresponding to the sequence of human p38 MAPK; final concentration 1/1000 dilution), MMP9 (Catalogue #2270; polyclonal antibody produced from rabbits immunized with synthetic peptide corresponding to amino acid residue Gly657 of human MMP9; final concentration 1/1000 dilution) and β-actin (Catalogue #4967; polyclonal antibody produced from rabbits immunized with synthetic peptide corresponding to amino-terminal residues of human β-actin; final concentration 1/1000 dilution) (all antibodies from Cell Signaling Technologies, Danvers, MA). Chemiluminescence detection was performed using the Bio-Rad Laboratories Molecular Imager ChemiDoc XRS+ imaging system. Densitometry was performed and represented as a ratio of pixel intensity of the phosphorylated p38 compared to total p38, using Bio-Rad Laboratories Image Lab software (version 4.0, build 16). Uncropped and unadjusted blots and gels are shown in S2 Fig. Gene expression was performed by quantitative PCR (qPCR) using validated Taqman probes (Life Technologies/Applied Biosystems, Carlsbad, CA). RNA was isolated using Qiagen RNeasy kit following tissue or cell homogenization and mRNA was reverse transcribed using the Applied Biosystems high capacity cDNA kit (Life Technologies). qPCR was performed on the Bio-Rad CFX384 real time system. No cDNA template, no reverse transcriptase treated samples and no DNA polymerase controls were examined for each qPCR throughout this study. Exogenous (human targets for mouse cDNA) and endogenous (β-actin) positive controls were also monitored for each assay. qPCR results are represented as relative quantification (RQ) compared to the mock treated animals or cells and corrected to β-actin levels.

### Statistical analyses

For statistical analysis, data from 10 animals or multiple separate cell experiments were pooled. Data are expressed as means ± S.E.M. Differences between groups of mice over time were compared by two-way analysis of variance (ANOVA) and multiple comparisons using the Bonferroni post-test. Pairs of groups were compared by Student’s t test (two tailed). p values for significance were set at 0.05. All analysis was performed using GraphPad Prism Software (Version 5 for Mac OS X).

## Results

### The induction of MMP9 during RSV infection prevents viral multiplicity

**Fig 1:**
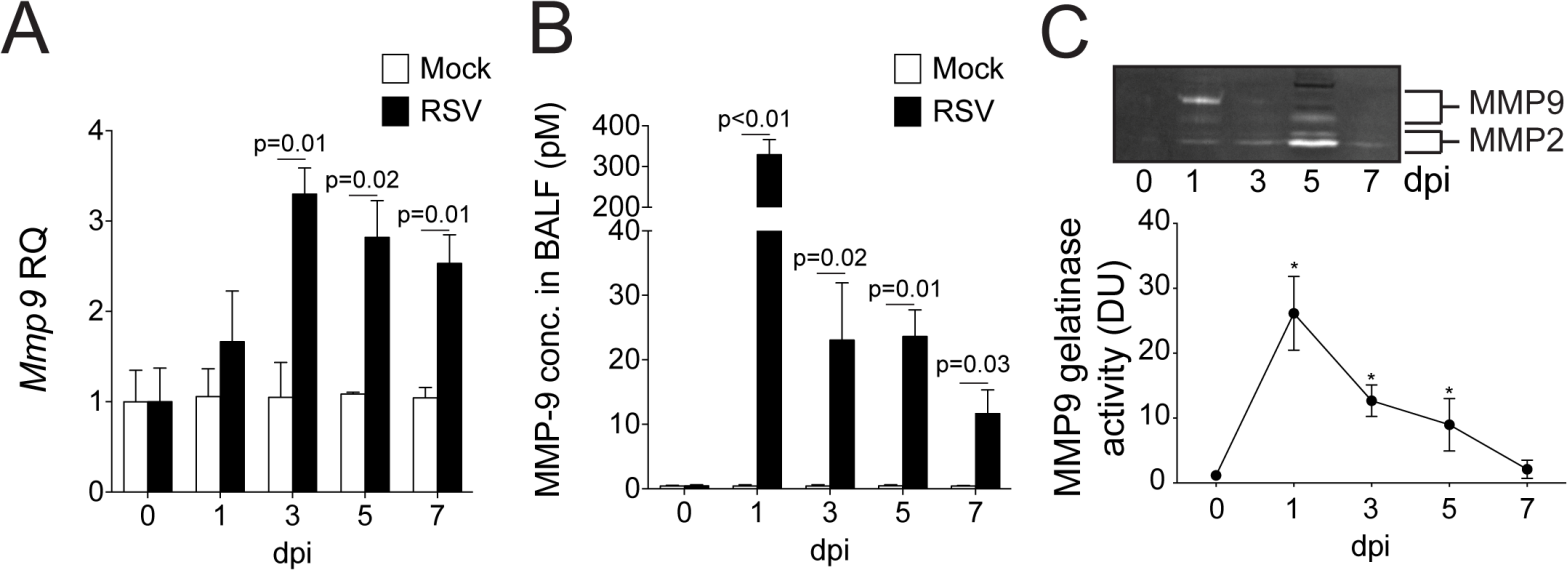
RSV infection induces host MMP9 expression and activity in mouse lungs. FVB/NJ mice were infected with 1x10^6^ pfu of RSV and a group of animals were euthanized at 0, 1, 3, 5 and 7 days post infection (dpi). (A) *Mmp9* lung gene expression was analyzed by qPCR. Graph are represented as relative quantification (RQ) of the mean ± S.E.M. (B) BALF MMP9 levels were determined by multiplex analysis on mock treated and RSV infected animals. (C) Gelatinase activity was analyzed in BALF using gelatin zymography. Bands corresponding to MMP-2 and MMP9 are highlighted and densitometry was performed for MMP9 bands from data pooled from 3 separate gels. Graphs are represented as mean ± S.E.M., where each measurement was performed 3 times on 10 animals/group. (A-B) p values shown, comparing both treatments connected by a line. (C) *Represents a p value less than 0.05 compared to mock treated mice on same day. All comparisons were determined by student t-tests.

Since increased MMP9 levels are observed during an RSV infection [12], we profiled MMP gene and protein expression, and activity in mice on multiple days during RSV infection. Mice exposed to RSV infection have increased lung MMP9 gene expression and elevated MMP9 protein and activity in the BALF (Fig 1). Gene transcription of *Mmp9* was significantly enhanced from 3 days post infection (Fig 1A). However, the most abundant BALF concentration and gelatinase activity of MMP9 occurs 1-day post RSV infection, with over 300 pM MMP9 observed in BALF (Fig 1B–1C).

**Fig 2:**
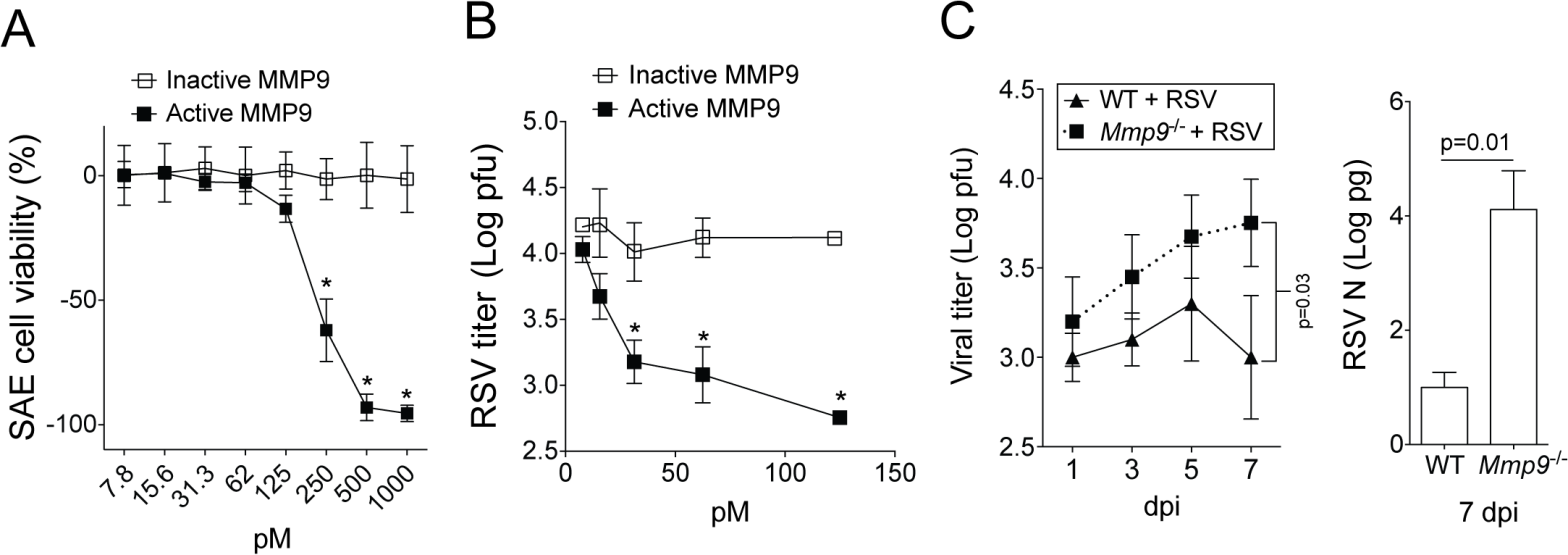
MMP9 prevents RSV infectivity of human airway epithelial cells and mouse lungs. (A) SAE cells were treated with various concentrations of active and inactive human MMP9 and TCID_50_ assays were performed 48 hours later. (B) RSV was treated with various concentrations of active and inactive MMP9 prior to infecting SAE cells. TCID_50_ assays were performed to determine the quantity of virus following MMP9 treatment. * Represents a p value less than 0.05 comparing inactive to active MMP9 for each concentration. All comparisons were determined by student t-tests. (C) *Mmp9* ^-/-^ mice and their FVB/NJ WT littermates were infected with 1x10^6^ pfu of RSV and animals were euthanized 1, 3, 5 and 7 dpi. Plaque assays and RSV N copy number, by qPCR (on 7 dpi), confirmed viral titers in lung tissue from all RSV-infected animals. Graphs are represented as mean ± S.E.M, with each measurement performed 3 times on 10 animals/group. Two-way ANOVA was used to compare the time-course curves and multiple comparisons were determined by the Bonferroni method (left panel). p value shown for qPCR (right panel) comparing both treatments connected by a line, determined by student t-tests.

To determine how the induction of MMP9 impacts on airway cells and RSV infectivity, SAE cells and RSV were exposed to either active or EDTA-inactivated MMP9 and viability assays were performed (Fig 2). RSV readily infects the airway epithelium [16], therefore human primary airway epithelial cells were investigated. SAE cells were utilized as they are not immortalized or cancerous in nature and secreted a similar cytokine profile to A549 cells during RSV infection [21]. At doses greater than 125 pM, active MMP9 significantly reduced the viability of SAE cells (Fig 2A). Sub-apoptotic levels of MMP9 (<125 pM) were incubated with RSV prior to infecting SAE cells to determine whether MMP9 could prevent RSV infectivity. Active MMP9 significantly reduced RSV viability determined by TCID_50_ assays (Fig 2B). The MMP9 levels observed to kill RSV are within the range frequently observed in RSV infections in infants [12] and during RSV-associated asthma exacerbations [25]. Viral load was examined in *Mmp9* ^*-/-*^ mice during RSV infection to confirm our *in vitro* data. Animals deficient for *Mmp9* had reduced clearance of RSV from the lungs, confirmed by plaque assays and qPCR (Fig 2C). Therefore lung MMP9 expression is required for viral clearance.

### *Mmp9* deficient mice experience heightened weight loss and AHR during RSV infection

**Fig 3:**
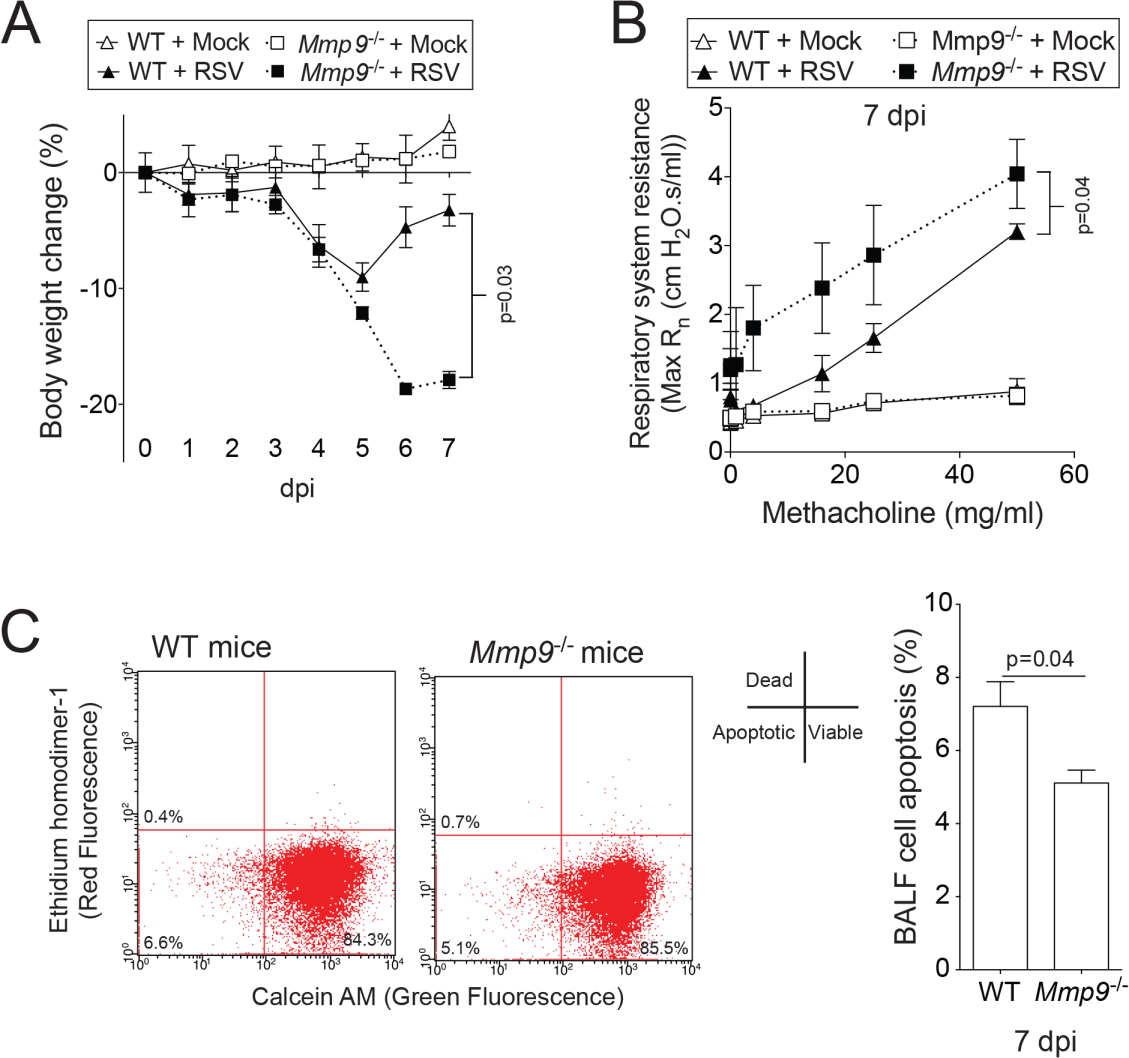
Loss of *Mmp9* expression enhances RSV infection-induced weight loss, AHR and reduces apoptosis in mice. *Mmp9* ^-/-^ mice and their FVB/NJ WT littermates were infected with 1x10^6^ pfu of RSV and animals were euthanized 7 dpi. (A) Animal body weight and (B) AHR to increasing doses of methacholine were assessed in each animal group 7 dpi. (C) BALF cells undergoing apoptosis were determined 7 dpi. Graphs are represented as mean ± S.E.M, with each measurement performed 3 times on 10 animals/group. Two-way ANOVA was used to compare the time-course curves and multiple comparisons were determined by the Bonferroni method (A and B). p values shown, comparing both treatments connected by a line by student t-tests (C).

MMP9 subdues AHR in asthma models [26, 27] but little is known about the impact of elevated concentrations of MMP9 on AHR during RSV infection. RSV-infected WT mice recovered from RSV infection-induced weight loss faster than their *Mmp9* ^-/-^ littermates (Fig 3A). The loss of *Mmp9* expression significantly enhanced RSV-induced AHR, demonstrated by respiratory system resistance (Rn) measurements during a methacholine dose challenge (Fig 3B). Loss of *Mmp9* expression reduced lung cell death, with BALF cells from *Mmp9* ^-/-^ mice undergoing apoptosis at a slower rate than from WT lungs, as determined by flow cytometry analysis (Fig 3C). These results demonstrate that MMP9 plays a major role in regulating AHR during RSV infection.

### Neutrophil infiltration is lower in *Mmp9* ^-/-^ mice

**Fig 4:**
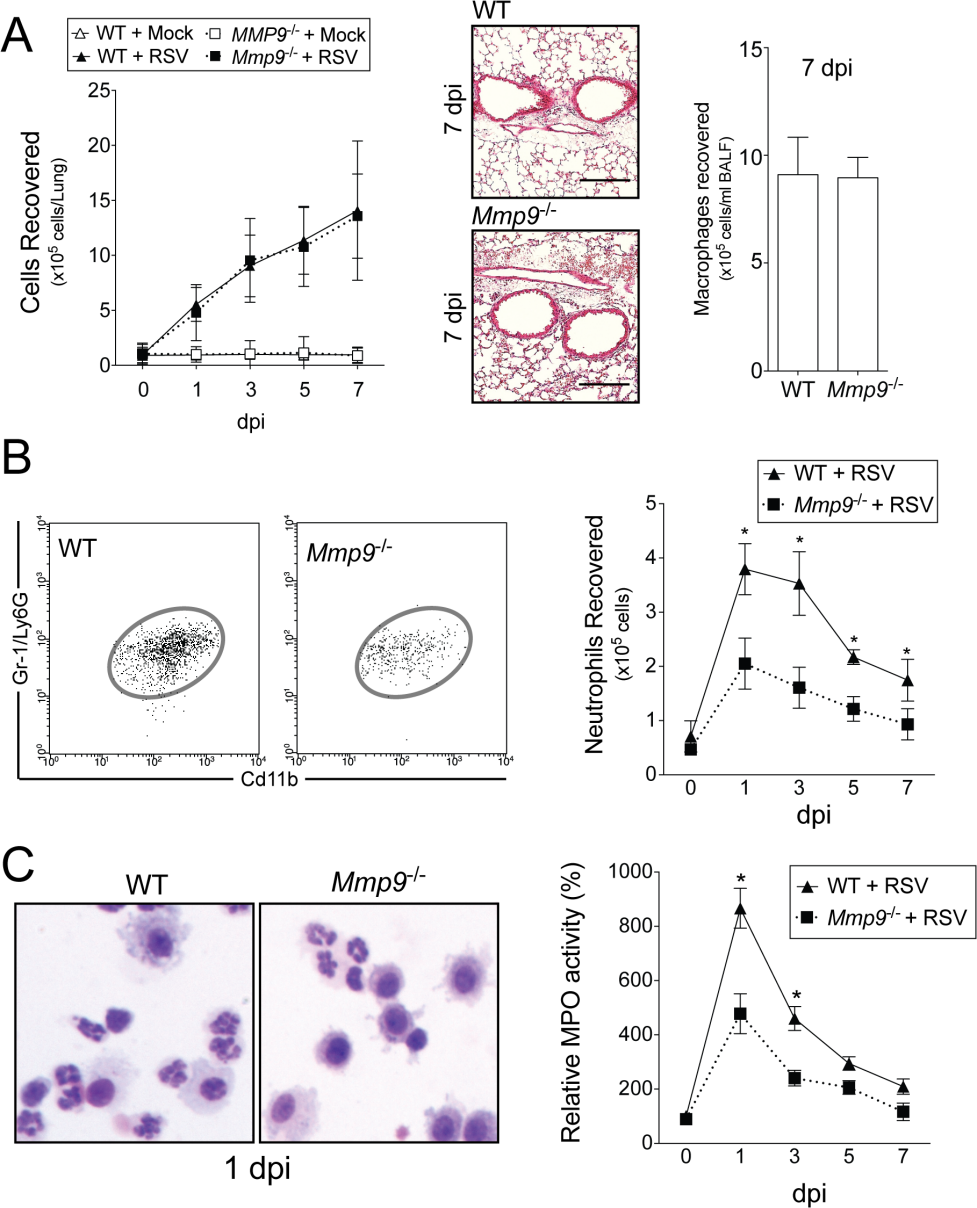
MMP9 expression regulates neutrophil recruitment to the lung during RSV infection. *Mmp9* ^-/-^ mice and their FVB/NJ WT littermates were infected with 1x10^6^ pfu of RSV and animals were euthanized 0, 1, 3, 5 and 7 dpi. (A) BALF total immune cellularity and alveolar macrophages (7 dpi) were determined. Comparative histology images of infected animals from each background are presented here (scale bar = 40 μm). (B) A typical representation of neutrophils from WT and *Mmp9* ^-/-^ mice 1 dpi of RSV, gated as SSC^high^CD11b^+^Gr-1^+^. Mean number of neutrophils from whole lung of WT and *Mmp9* ^-/-^ mice on days 0, 1, 3, 5, and 7 dpi. (C) Lung protein extracts were assayed for myeloperoxidase (MPO) activity from RSV-infected animals. A typical representation of BALF cells from WT and *Mmp9* ^-/-^ mice 1 dpi following cytospin and Diff-Quik staining. Graphs are represented as mean ± S.E.M, where each measurement was performed 3 times on 10 animals/group. Two-way ANOVA was used to compare the time-course curves and multiple comparisons were determined by the Bonferroni method. * Represents a p value less than 0.05 compared to WT mice on same day.

MMP9 is required for neutrophil migration during an influenza infection mouse model [10]. Therefore, we investigated the effect of MMP9 expression on lung neutrophils during RSV infection. RSV infection results in a significant increase in total BALF immune cell and alveolar macrophages that was not dependent on MMP9 expression (Fig 4A). Accumulation of neutrophils was observed from 1 dpi in the lung, as defined by flow cytometry for CD11b^high^Gr-1^high^ cells (Figs 4B and S1). *Mmp9* ^-/-^ mice had reduced neutrophil numbers present in the lung following infection (Fig 4B). To further quantify the effect of *Mmp9* deficiency on lung neutrophils, myeloperoxidase (MPO) activity levels were measured in lung tissue. The MPO activity in the lung tissue homogenate of *Mmp9* ^-/-^ mice was significantly less than WT mice (Fig 4C), which was consistent with the neutrophil influx. Neutrophils represented a large portion of the infiltrated cells in the BAL fluid at 24 hours in WT mice, further confirmed by Diff-Quik staining of immune cells after cytospin of the BALF (Fig 4C). These results confirm that MMP9 is crucial for neutrophil, but not macrophage, migration to the RSV infected lung.

### MMP9 regulates neutrophil chemotactic cytokines in RSV infected lungs

**Fig 5:**
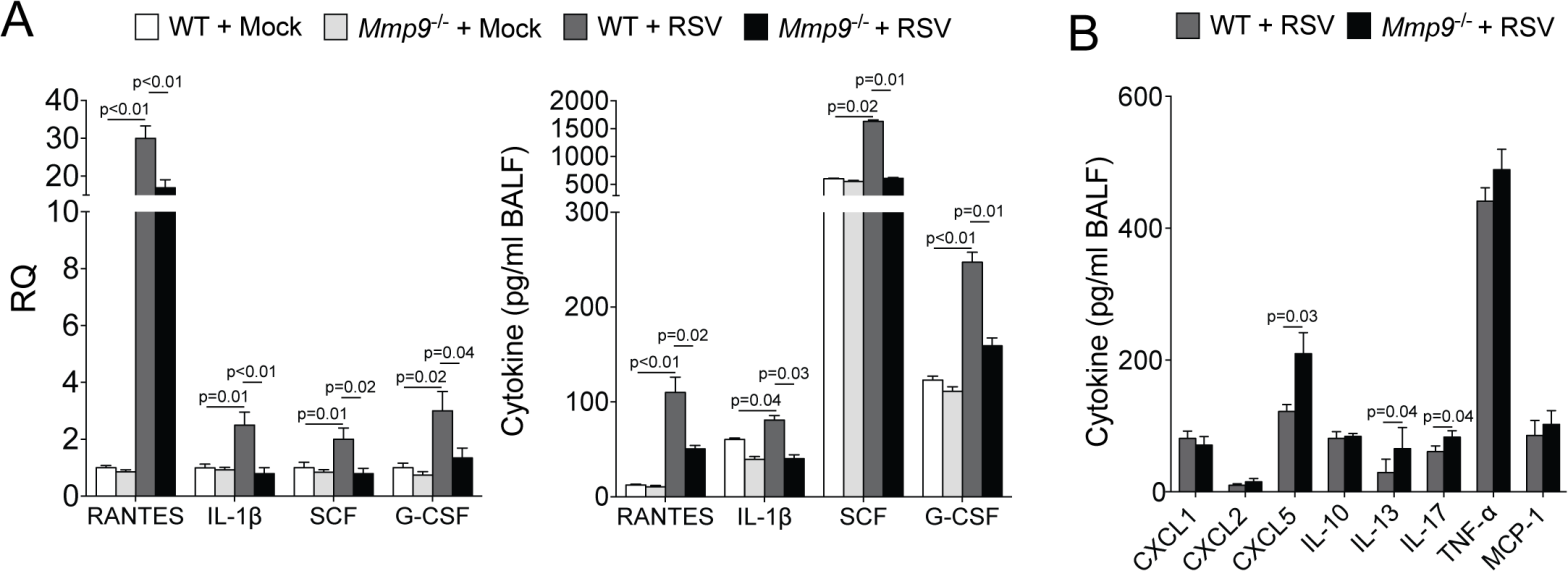
MMP9 regulates cytokine/chemokine expression during RSV infection. *Mmp9* ^-/-^ mice and their FVB/NJ WT littermates were infected with 1x10^6^ pfu of RSV and animals were euthanized 7 dpi. (A) Cytokine gene expression in lung tissue and BALF levels of RANTES, IL-1β, SCF and G-CSF were determined in both mouse genotypes, 7 dpi. (B) BALF levels of CXCL5, IL-13, CXCL2, TNF-α, MCP-1, IL-17, IL-10 and CXCL1 were determined in both mouse genotypes, 7 dpi. Graphs are represented as mean ± S.E.M, where each measurement performed 3 times on 10 animals/group. p values shown, comparing both treatments connected by a line. All comparisons were determined by student t-tests.

To investigate how MMP9 regulates neutrophil migration, we measured the expression and concentration of several cytokines associated with neutrophil recruitment in the lungs of WT and *Mmp9* ^-/-^ mice during RSV infection. Loss of *Mmp9* expression significantly reduced expression and secretion of RANTES, IL-1β, G-CSF and SCF in the lungs (Fig 5A). Interestingly, loss of *Mmp9* expression enhanced secretion of CXCL5, IL-13 and IL-17 following RSV infection compared to WT mice (Fig 5B) and these cytokines are associated with AHR [28]. MMP9 regulation of RANTES, IL-1β, G-CSF and SCF could regulate neutrophil recruitment to the lung during RSV infection.

**Fig 6:**
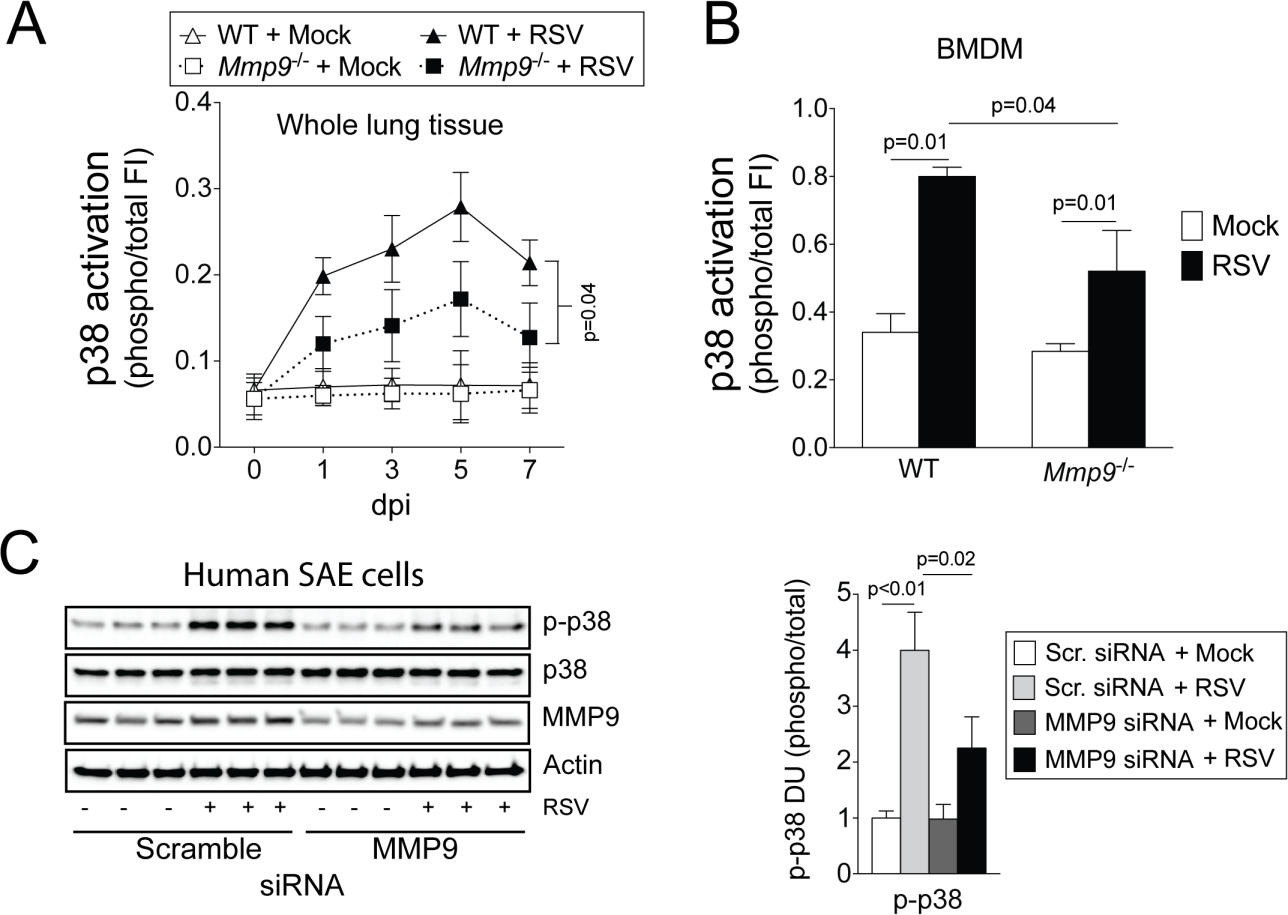
Loss of MMP9 expression blunts p38 responses to RSV infection. p38 activation was determined in (A) whole lung tissue and (B) bone marrow derived macrophages (BMDM) from WT and *Mmp9* ^-/-^ mice following RSV infection, using multiplex antibodies for the phosphorylated and total forms of each protein. (C) Immunoblots (p-p38, p38 and actin) were performed on SAE cells following MMP9 silencing and RSV infection for 24 hours. Densitometry analysis was determined. Densitometry units (DU) represent pixel intensity of phosphorylated p38 as a ratio of total p38. (A) Two-way ANOVA was used to compare the time-course curves and multiple comparisons were determined by the Bonferroni method. (B-C) p values shown comparing both treatments connected by a line, determined by student t-tests.

**Fig 7:**
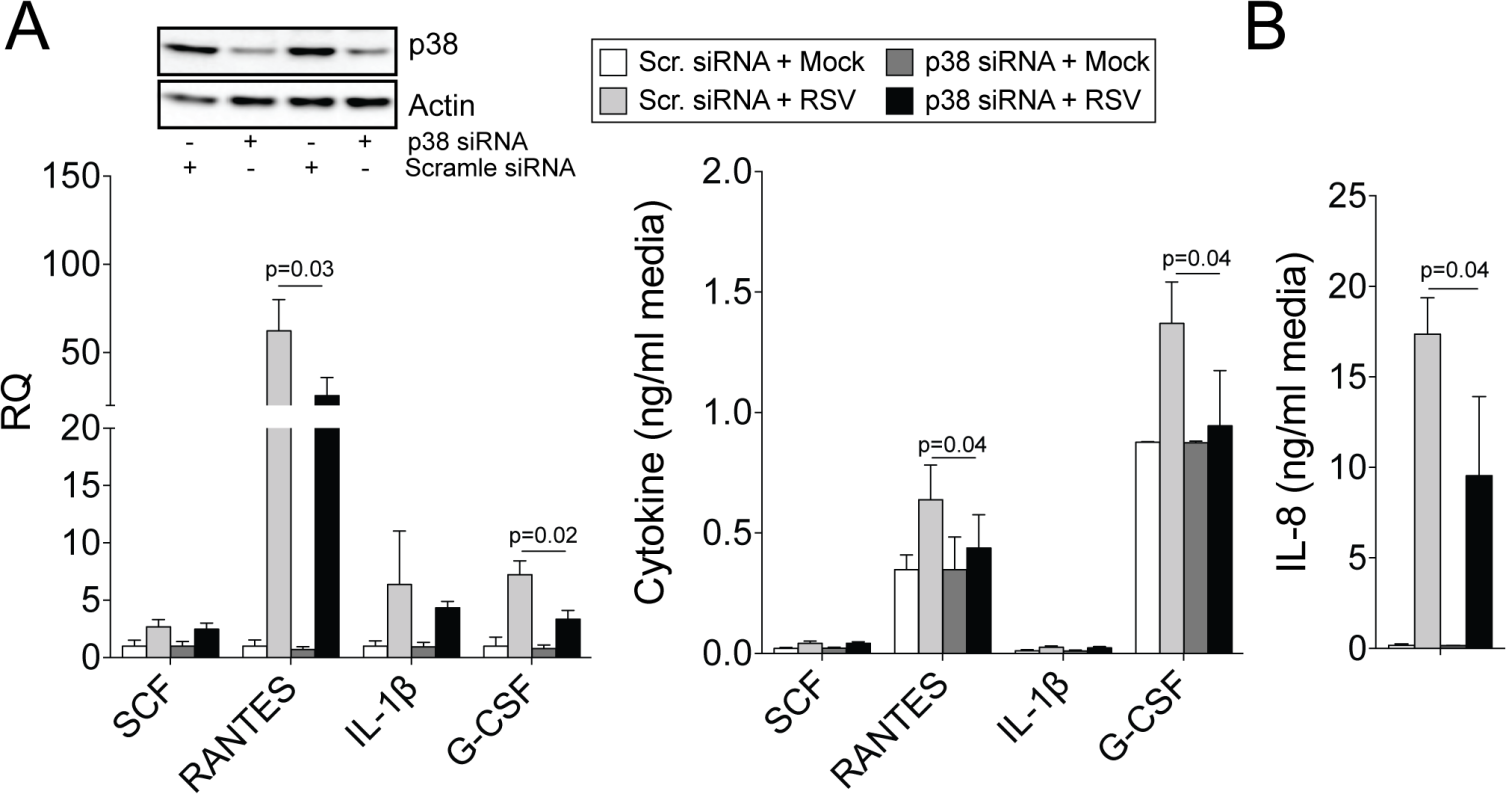
RANTES, G-CSF and IL-8 expressions are p38-dependent during RSV infection. SAE cells were transfected with p38 siRNA and infected with RSV for 24 hours. Immunoblots (p38 and actin) were performed on SAE cells to confirm reduced p38 protein translation. Cytokine gene expression and release from cells were analyzed for RANTES, IL-1β, G-CSF and SCF. IL-8 release was determined in cell media. Results are represented as relative quantification (RQ) and mean cytokine concentration ± S.E.M. Each measurement was performed 3 times on 6 replicates/group. p values shown, comparing both treatments connected by a line, determined by student t-tests.

Constitutive activation of the MAP-kinase p38 is critical for MMP9 production [29] but *Mmp9* expression was also critical for p38 phosphorylation in mouse lungs and BMDM from WT and *Mmp9* ^-/-^ mice (Fig 6A–6B). MMP9 was also observed to regulate p38 phosphorylation in human primary SAE cells following transfection of MMP9 siRNA (Fig 6C). Therefore, to determine whether reduced phosphorylation of p38 could regulate cytokines associated with neutrophil recruitment, p38 expression was silenced in SAE cells prior to RSV infection and MMP9-regulated cytokines (SCF, RANTES, IL-1β and G-CSF) were examined. Loss of p38 expression significantly reduced RANTES and G-CSF expression during RSV infection (Fig 7A). Both cytokines contribute to neutrophil recruitment to the lungs [30, 31]. IL-8 levels were also examined in human SAE cells, as mice do not express IL-8 [32] and IL-8 is a potent recruiter of neutrophils [33]. Loss of p38 resulted in reduced IL-8 production following RSV infection (Fig 7B). Therefore, MMP9 could act via p38 to mediate neutrophil recruitment by inducing the expression of RANTES, G-CSF and IL-8.

**Fig 8:**
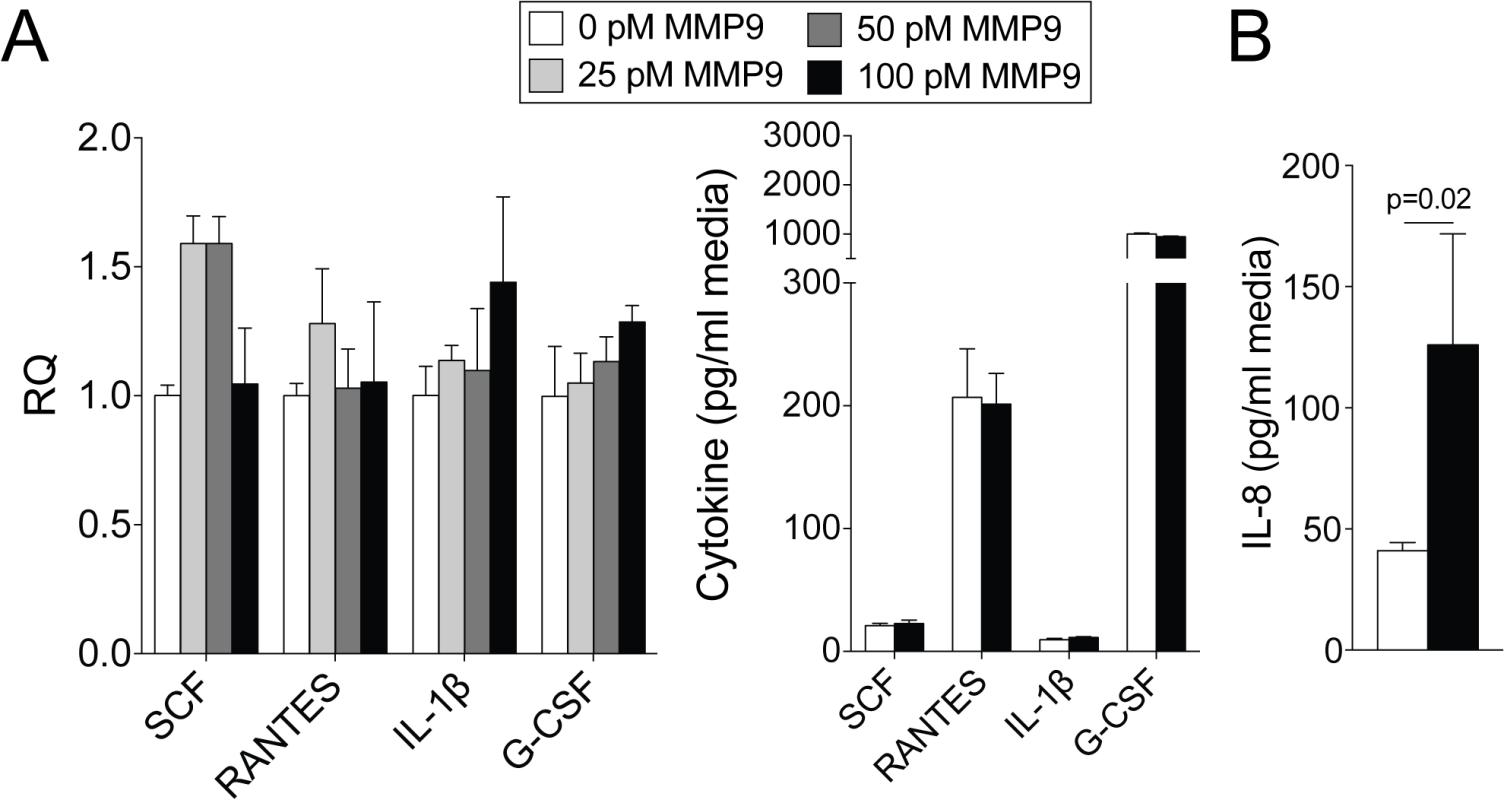
Extracellular active MMP9 induces IL-8 secretion from airway cells. SAE cells were treated with various concentrations of active and inactive human MMP9 for 24 hours. Cytokine gene expression and release from cells were analyzed for RANTES, IL-1β, G-CSF and SCF. IL-8 levels were determined in cell media. Results are represented as relative quantification (RQ) and mean cytokine concentration ± S.E.M. Each measurement was performed 3 times on 6 replicates/group. p value shown, comparing both treatments connected by a line, determined by student t-tests.

Since MMP9 expression regulates several cytokines that are associated with neutrophil recruitment to the lungs and MMP9 is present at high concentrations in the lung during RSV infection, we determined whether extracellular MMP9 could induce a cytokine response in SAE cells. Extracellular active MMP9 did not significantly impact on RANTES, IL-1β, G-CSF and SCF gene expression or secretion from SAE cells (Fig 8A). However extracellular MMP9 did induce IL-8 secretion (Fig 8B). Therefore, extracellular MMP9 can induce a cytokine response that could influence neutrophil migration to the lungs.

## Discussion

Though MMP9 levels correlate with RSV disease severity, its precise role in the pathogenesis of this infection has not previously been determined. Here, we identify several key functions of MMP9 during RSV infection. Prior to this study, RSV infection was known to elucidate a host MMP9 response [34] and we had predicted that MMP9 would play a major role in driving disease progression. However, this study demonstrates that host expression of MMP9 during RSV infection actually reduces RSV multiplicity and AHR. This study shows that MMP9 contributes neutrophil recruitment to the lungs during RSV infection. Furthermore, we identified that MMP9 triggers lung neutrophil infiltration possibly by enhancing p38 phosphorylation and subsequent RANTES, IL-8 and G-CSF expression during RSV infection.

Our study identifies a pronounced lung MMP9 response at day 1 post infection. In general, MMPs are rarely stored intracellularly and therefore require gene transcription before secretion. However MMP9 is constitutively present in neutrophil granules [35, 36]. The early MMP9 protein levels coincides with neutrophil infiltration in BALF and suggests neutrophil degranulation is a major source of MMP9 in early RSV infection. This observation is merited as the majority of the immune cells in human BALF from RSV patients are neutrophils [17] and depletion of neutrophils during an influenza infection mouse model significantly reduces airway MMPs [10]. Since neutrophils do not synthesize MMP-9 outside of the bone marrow [37], other cell types must contribute to the elevated gene expression of MMP9 later during RSV infection. These include a large number of differentiated cell types, including bronchial epithelial cells, monocytes, and macrophages [37–39]. Therefore we demonstrate that lung neutrophils are a significant source of MMP9 during RSV infection.

In recent years, the role of the neutrophil during RSV infection has been questioned. Though neutrophil-associated proteases play a critical role in the clearance of staphylococcal and candidal infections [40], lung neutrophilia is implicated in RSV infection severity [17]. Indeed, the number of neutrophils in BALF from RSV patients correlates positively with bronchiolitis severity [41] and lung neutrophil numbers decrease during RSV recovery in infants [17]. Others have demonstrated that MMP9, which is abundantly present in neutrophils, prevents AHR in asthma models [26, 27, 42, 43]. The ability of the neutrophil to cause AHR is well established [44]. However, what our data shows is that certain neutrophil components, specifically MMP9, actually counter AHR during RSV infection. Loss of MMP9 enhances IL-13 levels [27, 43], a cytokine implicated in the development of AHR [45] and the recruitment of eosinophils into the lungs [27, 46]. Therefore the role of neutrophils in RSV clearance and AHR still require further studies to determine their importance in RSV clearance and to extrapolate the relevance of the mouse findings to the human condition.

**Fig 9:**
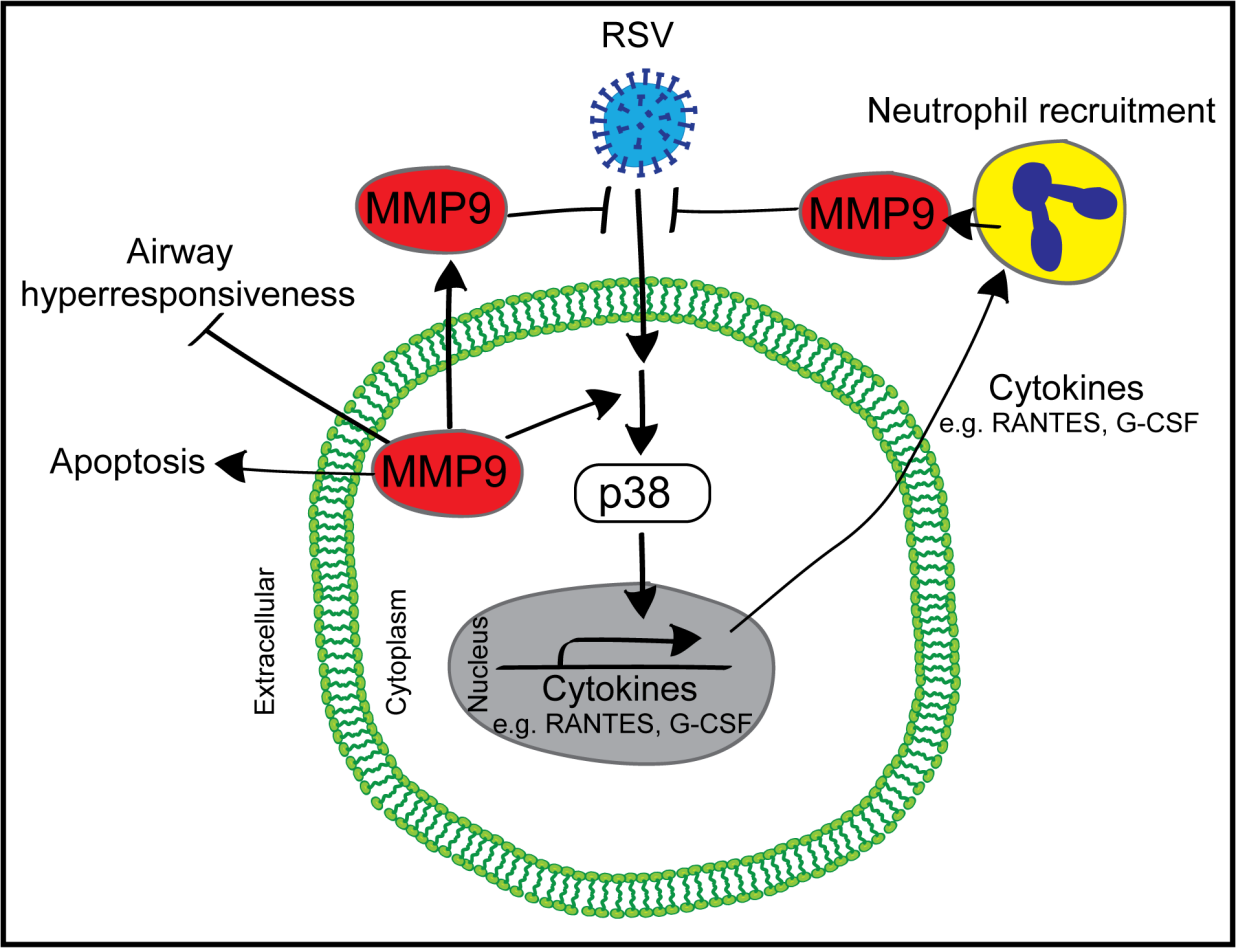
Proposed pathway for MMP9 signaling during RSV infection.

Loss of MMP9 expression had a significant impact on several cytokines and chemokines during RSV infection involved in the innate response of neutrophil recruitment. Loss of *Mmp9* expression during RSV infection resulted in increased levels of CXCL5, IL-17 and IL-13 and decreased RANTES, IL-1β, SCF and G-CSF compared to WT mice. Several of these targets are involved in neutrophil recruitment [47] and maturation [48]. Prior studies have established that the p38 pathway is critical for MMP9 expression [29] but here we determined that MMP9 impacts on p38 phosphorylation and on subsequent cytokine production as illustrated in Fig 9. In addition to upregulating cytokine production, MMP9 can cleave cytokines to alter their ability to recruit neutrophils. MMP9 cleaves CXCL5 thereby enhancing its chemotaxis ability, via binding to CXCR2 [47]. MMP-9 activities are absent in CXCR2^-/-^ mice, which have impaired neutrophil recruitment [49]. MMP9 could also regulate neutrophil recruitment via cleavage of extracellular matrix constituents, such as elastin and collagen [50]. These extracellular matrix fragments are potent chemoattractants [51], independent of cytokine responses [52].

MMP9 has the potential to injure tissues if released indiscriminately [2] and we observed enhanced apoptosis to epithelial cells following exposure to high concentrations of MMP9. We have identified an important role for MMP9 during RSV infection but other factors can lead to persistent elevated levels of MMP9 that could contribute to disease symptoms. MMP9 remains elevated in the sputum 6 months after smoking cessation, which might contribute to the continuous lung damage observed in COPD [53]. Overproduction of MMP9 following viral infection is associated with vascular leakage [54]. Therefore, the anti-viral potential of MMP9 comes at a cost to the host lungs with increased cell death, endothelial cell barrier disruption [54] and extracellular matrix degradation [2].

It was previously reported that inhibition of MMP9 activity prevented syncytia formation and blocked RSV multiplication *in vitro* [18]. However, the data here demonstrates that MMP9 reduces RSV infectivity *in vivo*. The earlier study by Yeo and colleagues utilized a protease inhibitor (1,10-phenanthroline) to block MMP9 [18] but 1,10-phenanthroline targets the activity of zinc-dependent MMPs, which could potentially impact on the activities of a substantial number of proteases. Recently, we identified that RSV infection triggers a large network of host proteases responses during RSV infection [13]. To this end, using a broad range inhibitor may have multiple non-specific effects on other zinc-activated proteases, proteins and ion channels.

The mechanisms by which MMP9 counters RSV infectivity *in vivo* remain to be determined. MMP9 could directly cleave proteins on RSV or the epithelial cell surface to inhibit viral attachment and entry. Alternatively, MMP9 could act indirectly to alter signaling processes that regulate antiviral responses and RSV replication. We believe MMP9 is exerting antiviral effects both directly and indirectly. MMP9 binds and interacts with several membrane proteins [55], such as epidermal growth factor (EGF) [56] and the surface-associated a2 (IV) chain of collagen IV [57]. Moreover, the active form of MMP9 binds to CD44 [58], a protein that co-localize with RSV proteins [59], but whether this interaction impacts on infectivity is unknown. CD44 expression is an indicative marker for effector-memory T-cells and CD44^-/-^ mice have reduced neutrophil emigration capabilities [60]. CD44 can also regulate p38 activation [61]. The role of p38 in RSV infectivity is intriguing; as inhibition of p38 activity can reduce infectivity [62] while RSV-induced sequestration of p38 into inclusion bodies reduces downstream signaling and cellular responses to RSV infection [63]. Therefore MMP-9 can associate with proteins on the cell surface of cells that regulate intracellular signaling, such as p38 phosphorylation, that counter RSV infectivity.

In summary, our studies demonstrate that MMP9 has anti-RSV properties that aid in viral clearance, neutrophil recruitment, and loss of MMP9 expression enhances disease manifestations. These findings provide important new insights into the role of MMP9 in innate responses to RSV infection.

## Supporting Information

S1 Fig An example of flow cytometry analysis with gating conditions for neutrophils. At least 25,000 events were acquired per sample. Multicolor flow cytometry analyses were used to evaluate the proportions of lung neutrophil populations. Figure shows raw data from one representative mouse with gating conditions. Granulocytes were gated from other cells as SSC^high^CD11b^+^ and Gr-1^high^.
(TIF)

S2 Fig Non-cropped pictures of blots. (A) Non-cropped picture of zymogram gel presented in Fig 1C. Non-cropped pictures of blots presented in Fig (B) 7C and (C) 8.
(TIF)
