# Supplementary figures and images for: Matrix Metalloproteinase 9 Exerts Antiviral Activity against Respiratory Syncytial Virus
Source: PLoS One. 2015 Aug 18;10(8):e0135970. doi: 10.1371/journal.pone.0135970 (PMC4540458; doi:10.1371/journal.pone.0135970)

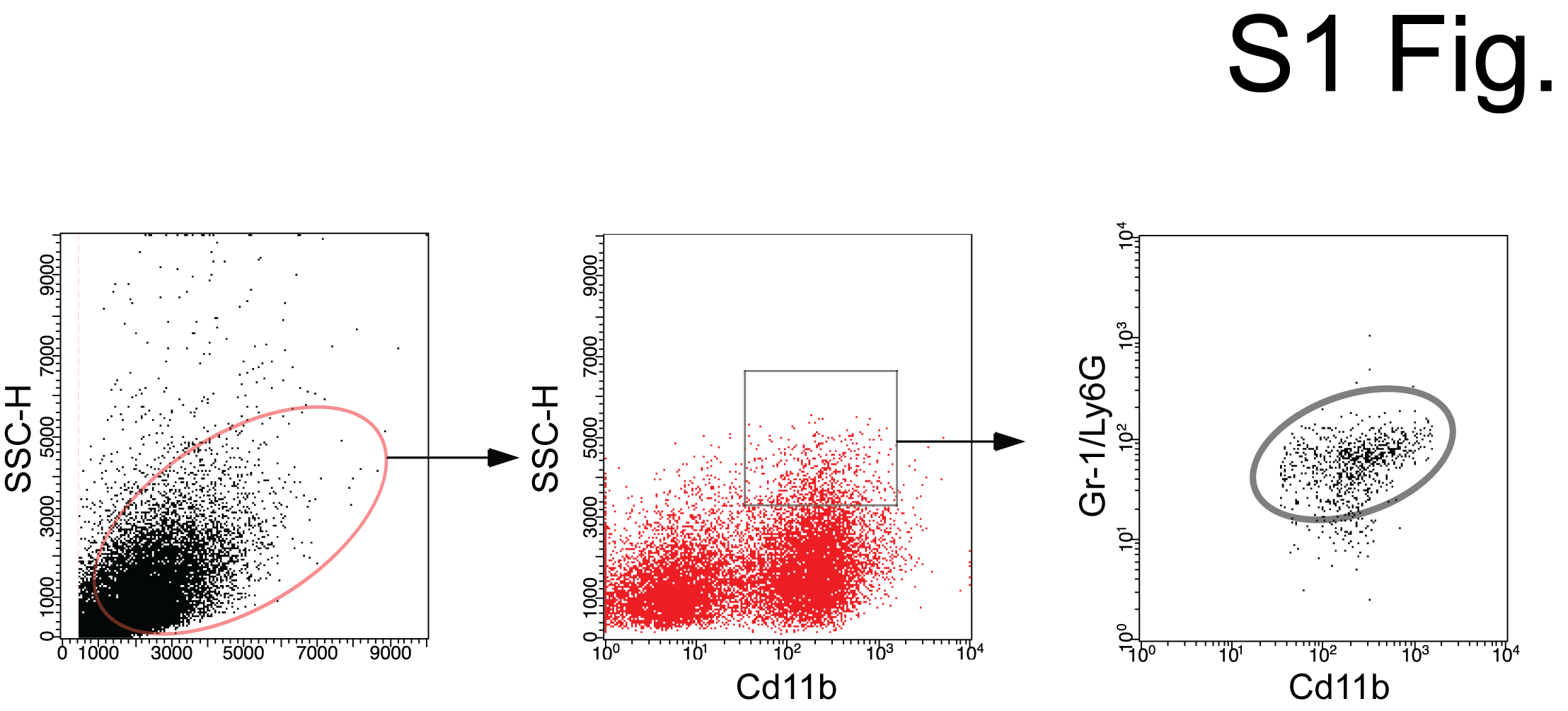

Supplement: S1 Fig — At least 25,000 events were acquired per sample. Multicolor flow cytometry analyses were used to evaluate the proportions of lung neutrophil populations. Figure shows raw data from one representative mouse with gating conditions. Granulocytes were gated from other cells as SSChighCD11b+ and Gr-1high. (TIF) [file pone.0135970.s001.tif]

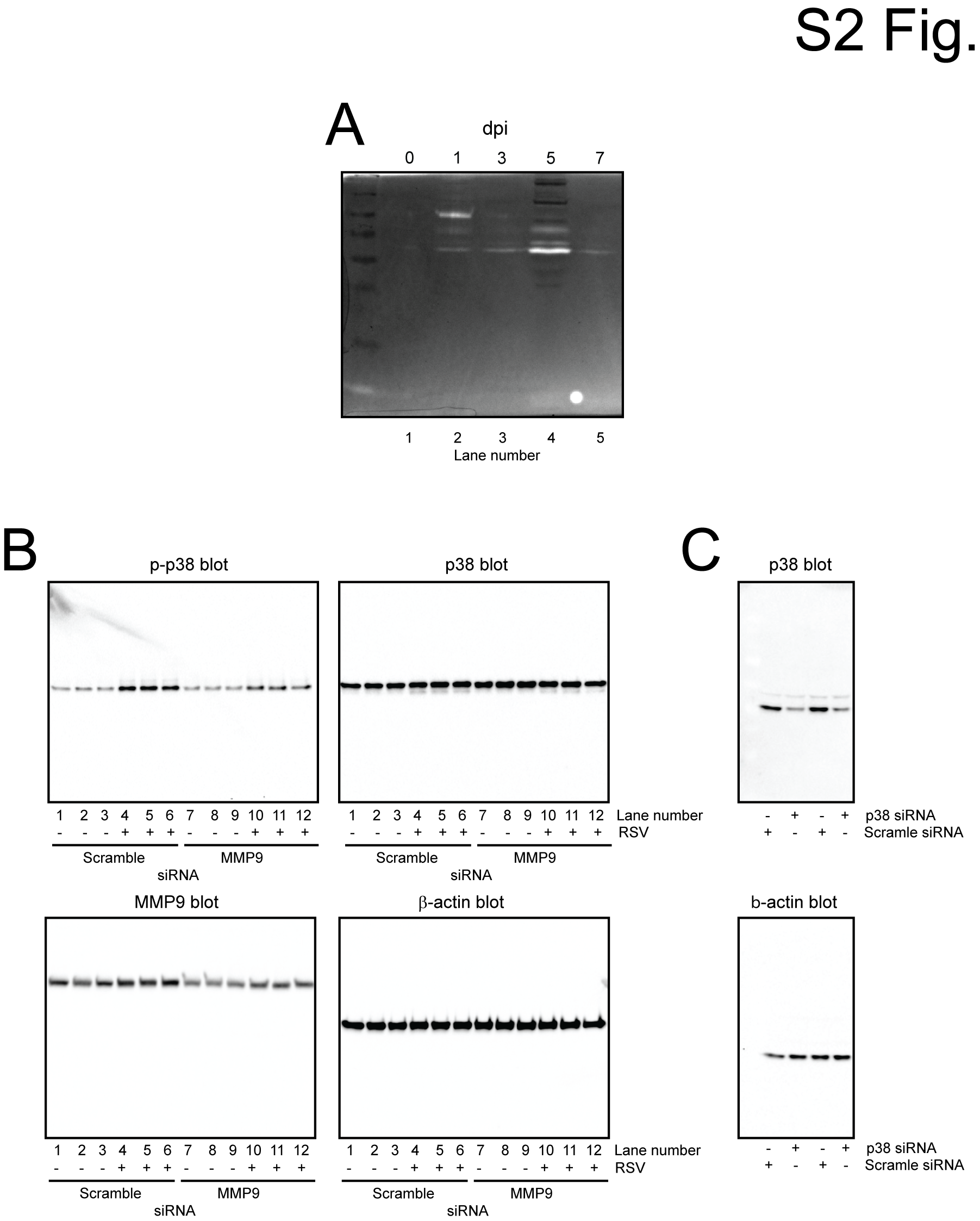

Supplement: S2 Fig — (A) Non-cropped picture of zymogram gel presented in Fig 1C. Non-cropped pictures of blots presented in Fig (B) 7C and (C) 8. (TIF) [file pone.0135970.s002.tif]
